# Long noncoding RNA expression signature to predict platinum-based chemotherapeutic sensitivity of ovarian cancer patients

**DOI:** 10.1038/s41598-017-00050-w

**Published:** 2017-02-02

**Authors:** Rong Liu, Ying Zeng, Cheng-Fang Zhou, Ying Wang, Xi Li, Zhao-Qian Liu, Xiao-Ping Chen, Wei Zhang, Hong-Hao Zhou

**Affiliations:** 1 Department of Clinical Pharmacology, Xiangya Hospital, Central South University, Changsha, 410008 P. R. China; 2 Institute of Clinical Pharmacology, Central South University; Hunan Key Laboratory of Pharmacogenetics, Changsha, 410078 P. R. China; 3 The Affiliated Cancer Hospital of XiangYa School of Medicine, Central South University, Changsha, Hunan 410014 P. R. China

## Abstract

Dysregulated long noncoding RNAs (lncRNAs) are potential markers of several tumor prognoses. This study aimed to develop a lncRNA expression signature that can predict chemotherapeutic sensitivity for patients with advanced stage and high-grade serous ovarian cancer (HGS-OvCa) treated with platinum-based chemotherapy. The lncRNA expression profiles of 258 HGS-OvCa patients from The Cancer Genome Atlas were analyzed. Results revealed that an eight-lncRNA signature was significantly associated with chemosensitivity in the multivariate logistic regression model, which can accurately predict the chemosensitivity of patients [Area under curve (AUC) = 0.83]. The association of a chemosensitivity predictor with molecular subtypes indicated the excellent prognosis performance of this marker in differentiated, mesenchymal, and immunoreactive subtypes (AUC > 0.8). The significant correlation between ZFAS1 expression and chemosensitivity was confirmed in 233 HGS-OvCa patients from the Gene Expression Omnibus datasets (GSE9891, GSE63885, and GSE51373). *In vitro* experiments demonstrated that the ZFAS1 expression was upregulated by cisplatin in A2008, HeyA8, and HeyC2 cell lines. This finding suggested that ZFAS1 may participate in platinum resistance. Therefore, the evaluation of the eight-lncRNA signature may be clinically implicated in the selection of platinum-resistant HGS-OvCa patients. The role of ZFAS1 in platinum resistance should be further investigated.

## Introduction

Ovarian cancer yields the highest mortality rate of all lethal gynecologic cancers and represents approximately 3% of all cancers diagnosed in women worldwide^1,2^. The prognosis of ovarian cancer is unsatisfactory, with a 5-year survival rate of approximately 30%^3^. Approximately 70% of patient deaths are advanced stage and high-grade serous ovarian cancers (HGS-OvCa)^4^. Despite advancements in surgery and chemotherapy, platinum-resistant cancer recurs in approximately 25% of patients within 6 months after they undergo initial standard treatments consisting of aggressive surgery and platinum-based chemotherapy^5^. Some patients with a complete response to first-line chemotherapy develop acquired drug resistance^6^. Several molecular mechanisms, including drug efflux and tolerance, increased DNA repair, and increased cellular glutathione levels^7–9^, are implicated in chemosensitivity. However, exact mechanisms have yet to be fully investigated. Clinical biomarkers that accurately predict sensitivity to chemotherapy have yet to be developed^10,11^. These factors should be understood to identify prognostic signatures, which can be utilized to develop effective treatment modalities for stratified patients who unlikely respond to platinum-based chemotherapy and thus can benefit from alternative strategies^10^.

Dysregulated and functional long noncoding RNAs (lncRNAs) are associated with the tumorigenesis and progression of various human cancers^12–14^. lncRNAs are mRNA-like transcripts range from 200 nucleotides (bp) to multiple kilobases (kb) in length but lack a coding capacity^15^. In ovarian cancer, some dysregulated lncRNAs function as tumor suppressor genes, proto-oncogenes, and metastatic transformation stimulator^16–22^. Increased HOTAIR, AB073614, and CCAT2 expression levels are associated with poor prognosis and high metastatic probability^16,17,20^. LSINCT5 is overexpressed in ovarian cancer cell lines and tumor tissues and implicated in the cellular proliferation and development of ovarian cancer^18^. The downregulation of BC200 in ovarian cancer is involved in cancer cell proliferation and mediation of carboplatin-induced cancer cell death^19^. Zhou *et al.* ^21^ identified an eight-lncRNA signature that can be used to classify patients with poor and improved overall survival rates. Two immune-related lncRNAs, namely, RP11-284N8.3.1 and AC104699.1.1, have been identified as predictors of an ovarian cancer patient’s survival rates by using lncRNA–mRNA coexpression network methods^22^. Similar to protein-coding genes and miRNAs, lncRNAs can be utilized as biomarkers for diagnosis and prognosis. However, the prognostic significance of lncRNAs in the chemotherapeutic sensitivity of HGS-OvCa treated with platinum-based chemotherapy has yet to be investigated.

In this study, the association between lncRNA expression profiles and platinum-based chemotherapy sensitivity for HGS-OvCa patients from the Cancer Genome Atlas (TCGA) Research Network was investigated to determine whether lncRNA expression profiling can be used as a prognostic predictive signature for chemotherapeutic sensitivity. Our findings were validated on the basis of independent datasets from Gene Expression Omnibus (GEO).

## Results

### Identification of lncRNAs from the training sets

**Figure 1:**
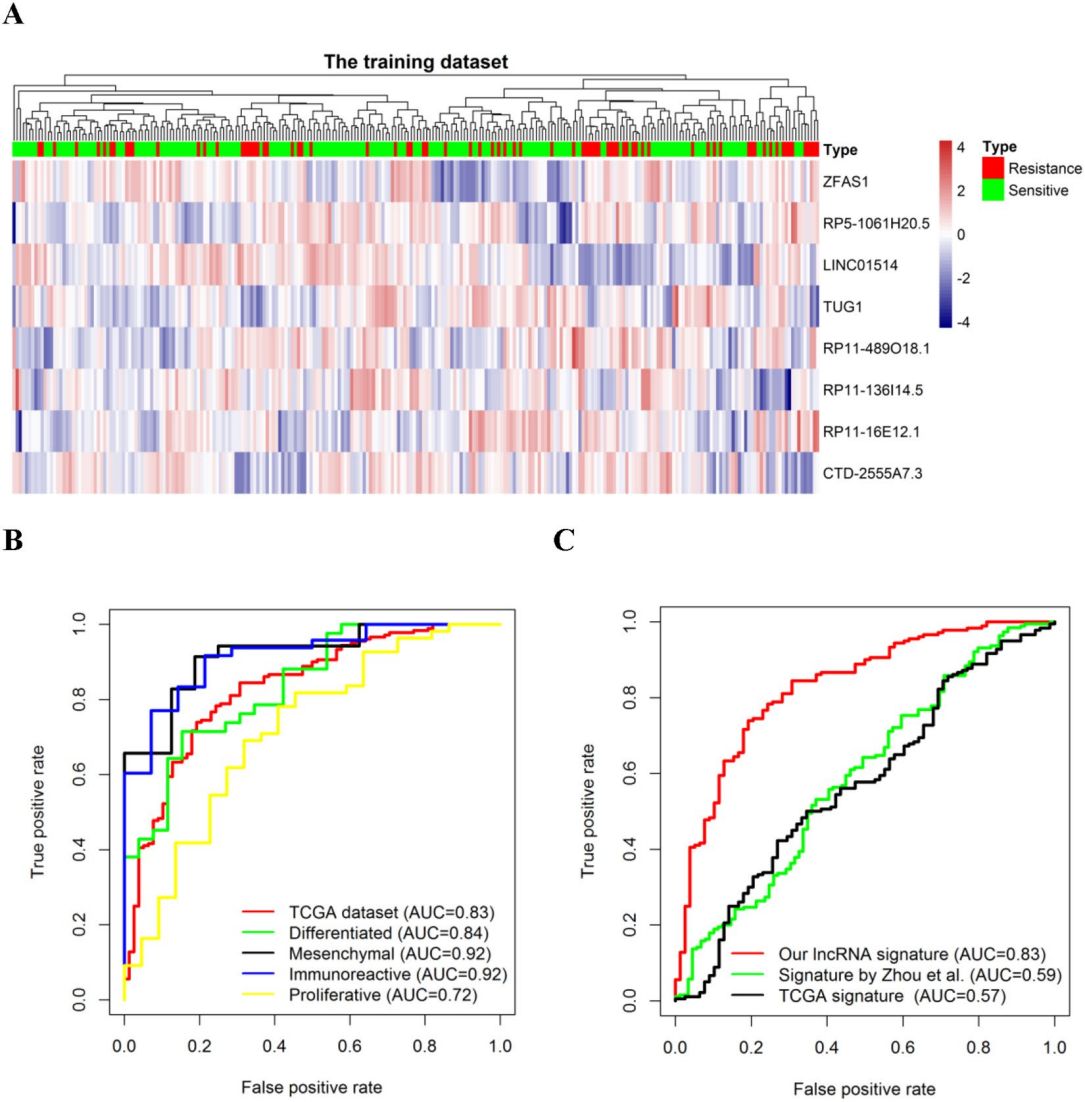
Unsupervised clustering heatmap and ROC curves for the eight- lncRNA signature. Heatmap based on eight lncRNAs (rows) of HGS-OvCa patients (columns) in the TCGA datasets (n = 258). Red and blue indicate high and low expression levels, respectively (**A**). ROC curves represent the accuracy of the eight-lncRNA signature in the TCGA dataset and different subtypes (**B**), and ROC curves represent the accuracy of our defined signature, the lncRNA signature developed by Zhou *et al.*, and the TCGA mRNA prognostic signature (**C**). True positive rate represents sensitivity, whereas false positive rate is one minus the specificity.

The TCGA dataset with 258 HGS-OvCa patients was used for the detection of lncRNAs related with platinum chemotherapeutic sensitivity. By subjecting the lncRNA expression data to univariate and multivariate logistic regression models, we identified a set of eight lncRNAs that were significantly correlated with the patients’ chemotherapeutic sensitivity (p < 0.003 in the univariate model and p < 0.01 in the multivariate model; Table 1). The higher expression levels of ZFAS1, RP5-1061H20.5, RP11-489O18.1, and RP11-16E12.1 were associated with the lower probability of chemotherapeutic sensitivity (OR < 1 in both the univariate and multivariate models). On the other hand, the higher expression levels of LINC01514, TUG1, RP11-136I14.5, and CTD-2555A7.3 were associated with the higher probability of chemotherapeutic sensitivity (OR > 1 in both the univariate and multivariate models) (Fig. 1A and Table 1). The complete list of lncRNAs that were associated with the patients’ chemotherapeutic sensitivity with p < 0.05 in the univariate model of the training dataset is shown in Table S1.

**Table 1:** Logistic regression model for chemosensitive patients with complete clinical and genomic data in the training dataset (n = 258).

Gene id	Gene symbol	Chromosome	Univariate model			Multivariate model		
			OR	95% CI	P value	OR	95% CI	P value
ENSG00000177410.12	ZFAS1	chr20: 49278178–49295738 (+)	0.65	0.49–0.85	2.74 × 10^−3^	0.61	0.43–0.87	6.72 × 10^−3^
ENSG00000233920.1	RP5-1061H20.5	chr1: 229223461–229227562 (−)	0.59	0.43–0.79	7.57 × 10^−4^	0.53	0.37–0.74	3.36 × 10^−4^
ENSG00000237579.2	LINC01514	chr10: 101176323–101194147 (+)	1.56	1.19–2.06	1.64 × 10^−3^	1.78	1.28–2.53	8.19 × 10^−4^
ENSG00000253352.8	TUG1	chr22: 30970677–30979395 (+)	1.64	1.24–2.20	7.10 × 10^−4^	1.67	1.19–2.41	4.23 × 10^−3^
ENSG00000253988.1	RP11-489O18.1	chr8: 138063268–138073240 (+)	0.63	0.47–0.83	1.19 × 10^−3^	0.60	0.42–0.83	3.13 × 10^−3^
ENSG00000255689.1	RP11-136I14.5	chr11: 115582297–115600339 (+)	1.61	1.22–2.18	1.14 × 10^−3^	1.72	1.25–2.43	1.34 × 10^−3^
ENSG00000259448.2	RP11-16E12.1	chr15: 31216020–31224445 (+)	0.64	0.48–0.85	2.44 × 10^−3^	0.57	0.40–0.81	2.04 × 10^−3^
ENSG00000261546.1	CTD-2555A7.3	chr16: 89113175–89115279 (−)	1.51	1.16–2.00	2.77 × 10^−3^	1.61	1.17–2.26	4.33 × 10^−3^

### Eight-lncRNA signature and chemotherapeutic sensitivity

We created a risk-score formula according to the expression levels of eight lncRNAs for the chemotherapeutic sensitivity prediction as follows: predictive score = (−0.4410 × expression level of ZFAS1) − (0.6380 × expression level of RP5-1061H20.5) + (0.5775 × expression level of LINC01514) + (0.5143 × expression level of TUG1) − (0.5167 × expression level of RP11-489O18.1) + (0.5425 × expression level of RP11-136I14.5) − (0.5595 × expression level of RP11-16E12.1) + (0.4771 × expression level of CTD-2555A7.3). According to this risk score, patients in the training set were divided into low-score and high-score groups using the median risk score as the cut-off. The high-score group showed a higher probability of sensitivity (OR = 9.06, 95% CI = 4.77–18.35, p = 1.07 × 10^−10^ in the univariate model; OR = 9.58, 95% CI = 4.97–19.73, p = 1.05 × 10^−10^ in the multivariate model). In addition, ROC analysis was performed to assess the predictive accuracy of the eight-lncRNA signature. The lncRNA signature showed a predictive power in distinguishing sensitive from resistance either in the training dataset (AUC = 0.83, Fig. 1B) or in different molecular subtypes (AUC > 0.7, Fig. 1B). Furthermore, compared with the two published tests (signature by Zhou *et al.* and TCGA), our defined lncRNA signature showed a better performance as demonstrated by higher AUC values (Fig. 1C).

### Eight-lncRNA signature and chemotherapeutic response

**Figure 2:**
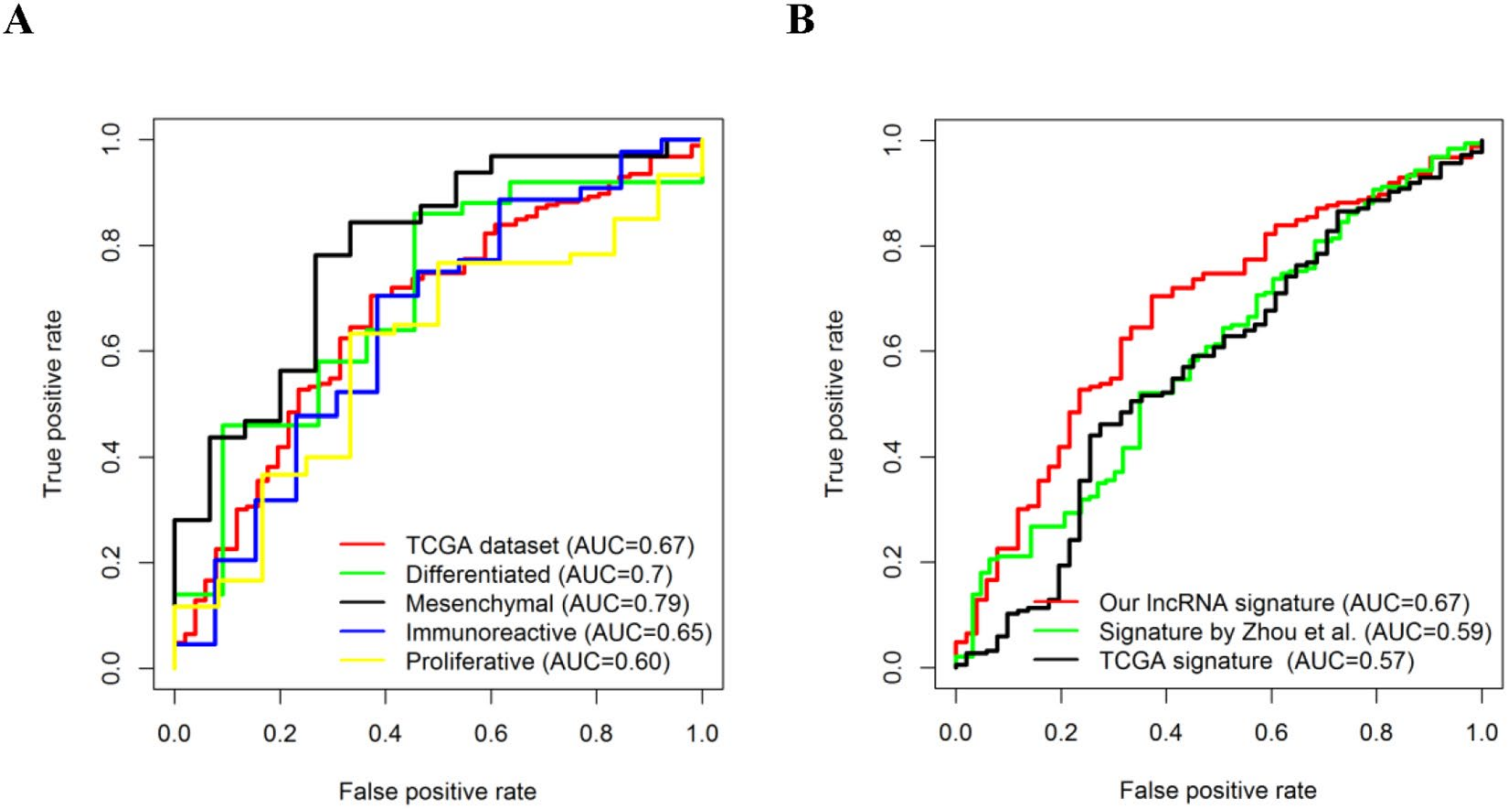
ROC curves for the eight-lncRNA signature in predicting chemoresponses. ROC curves represents the accuracy of the lncRNA signature in the training dataset and different subtypes in predicting chemoresponses (**A**), and the accuracy of our defined lncRNA signature, the lncRNA signature developed by Zhou *et al.*, and the TCGA mRNA prognostic signature (**B**). True positive rate represents sensitivity, whereas false positive rate is one minus the specificity.

In addition to the association of chemotherapeutic sensitivity in ovarian cancer, the significant associations between the identified eight-lncRNA signature and chemotherapeutic response were also investigated. ROC analysis showed that our defined lncRNA signature was predictive of a complete response in the whole training dataset (AUC = 0.67) and across different subtypes (AUC ≥ 0.6, Fig. 2A). In addition, the eight-lncRNA signature showed higher AUC values than the two published signatures developed by Zhou *et al.* and the TCGA group (Fig. 2B).

### Prognostic value of the eight-lncRNA signature that is independent of clinical information

The multivariate logistic regression analysis was conducted to confirm whether the eight-lncRNA expression signature was an independent predictor of HGS-OvCa patients’ sensitivity after platinum-based chemotherapy. In the model, chemotherapeutic sensitivity was a dependent variable, and stage, grade, molecular subtypes, and lncRNA predictive score were covariates. Specifically, results showed that the eight-lncRNA signature is an independent predictor of chemotherapeutic sensitivity when adjusted using the above-mentioned covariates (OR = 9.58, 95% CI = 4.97–19.73; p = 1.05 × 10^−10^) (Table 2).

**Table 2:** Univariable and multivariable logistic regression models in the training dataset.

	Univariate model			Multivariable model		
	OR	95% CI	P value	OR	95% CI	P value
**LncRNA signature (high/low)**	9.06	4.77–18.35	1.07 × 10^−10^	9.58	4.97–19.73	1.05 × 10^−10^
Stage (ref = 2)						
3	0.16	0.01–0.85	8.38 × 10^−2^	0.16	0.01–1.03	1.05 × 10^−1^
4	0.18	0.01–1.13	1.29 × 10^−1^	0.16	0.01–1.19	1.20 × 10^−1^
Grade (ref = 2)						
3	0.65	0.26–1.44	3.10 × 10^−1^	0.69	0.25–1.78	4.53 × 10^−1^
Molecular subtypes (ref = differentiated)						
Immunoreactive	2.12	0.99–4.68	5.46 × 10^−2^	2.51	1.06–6.14	3.94 × 10^−2^
Mesenchymal	1.35	0.63–2.95	4.39 × 10^−1^	1.63	0.68–3.96	2.76 × 10^−1^
Proliferative	1.55	0.77–3.12	2.18 × 10^−1^	1.45	0.64–3.30	3.68 × 10^−1^

### LncRNA ZFAS1 association with chemotherapeutic sensitivity in ovarian cancer subtypes

**Figure 3:**
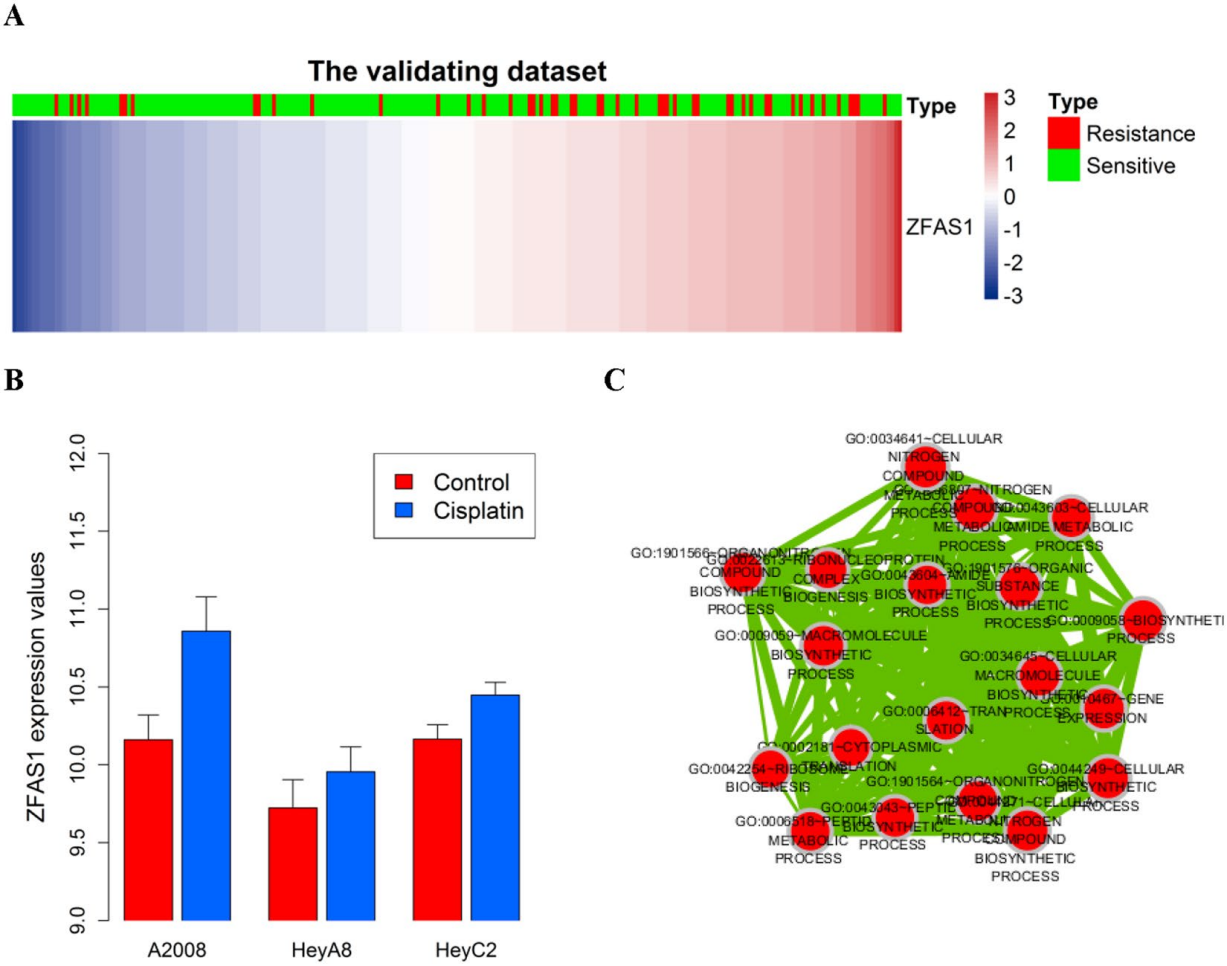
Associations between ZFAS1 and chemosensitivity are observed in the validating datasets. Heatmap based on the genes (rows) of patients with ovarian cancer (columns) for the ZFAS1 in the validating dataset (**A**). Red and blue indicate high and low expression levels, respectively. The expression values of ZFAS1 in A2008, HeyA8, and HeyC2 cell lines treated with or without cisplatin treatment. p values were calculated by independent two-tailed t test. Error bars represent the mean ± SD (**B**). The functional map of enriched GO terms with each node indicates an enriched GO term, and each edge represents the common genes shared between connecting and enriched GO terms (**C**).

Among the eight-lncRNAs, only three lncRNAs (ZFAS1, LINC01514, and TUG1) were observed in the validation dataset, and the role of ZFAS1 was confirmed in the validation dataset (OR = 0.67, 95% CI = 0.48–0.94; p = 2.12×10^−2^, Fig. 3A). The probe name of ZFAS1 by Affymetrix U133 Plus 2 platform is 224915_x_at. In addition to the association of chemotherapeutic sensitivity in ovarian cancer, the associations between ZFAS1 and molecular subtypes were also studied. Results show that the increased expression level of ZFAS1 can be accomplished with a low probability of sensitivity for all subtypes (OR < 1). However, accounting for the small sample size within molecular subtypes, the relationship between ZFAS1 and probability of sensitivity is only statistically significant in the training dataset (OR = 0.58, p value = 4.83 × 10^−2^) of differentiated subtypes (Table 3).

**Table 3:** Relationship between ZFAS1 with chemosensitivity in ovarian cancer molecular subtypes.

Molecular subtype	Training dataset			Validating dataset		
	OR	95% CI	P value	OR	95% CI	P value
Proliferative	0.58	0.32–0.97	4.83 × 10^−2^	0.53	0.24–1.04	8.65 × 10^−2^
Mesenchymal	0.64	0.33–1.20	1.71 × 10^−1^	0.69	0.21–2.00	5.18 × 10^−1^
Differentiated	0.59	0.30–1.04	8.76 × 10^−2^	0.75	0.38–1.45	3.93 × 10^−1^
Immunoreactive	0.71	0.36–1.35	3.12 × 10^−1^	0.61	0.28–1.22	1.82 × 10^−1^

### LncRNA ZFAS1 may be associated with platinum resistance

From the above-mentioned results, we can conclude that the high expression level of ZFAS1 correlate with low sensitivity in HGS-OvCa patients treated with platinum, suggesting that ZFAS1 might be associated with platinum resistance. To validate this hypothesis, GSE47856^23^ was downloaded from GEO and analyzed. The probe that corresponded to ZFAS1 by Human Gene ST 1.0 arrays was 8063337. *In vitro* experiment results showed that the ZFAS1 expression level was upregulated in A2008, HeyA8, and HeyC2 cell lines treated with cisplatin compared with the control group (Fig. 3B), which indicates that cisplatin could increase the ZFAS1 expression level in ovarian cancer cells. Results for the 17 cell lines with at least three replicates are illustrated in Table S2.

### Functional annotation

The coexpressed relationships between the expression levels of eight lncRNAs and protein-coding genes (PCGs) were investigated by determining Pearson’s correlation coefficients in the TCGA dataset to further investigate the potential biological roles involving the prognostic lncRNA biomarkers. The expression level of 24 PCGs was highly correlated with that of ZFAS1 (R ≥ 0.4, Table S3). Gene ontology (GO) function enrichment analysis of these PCGs was then performed with the whole human genome as the background. GO functional annotation suggested that these PCGs were significantly enriched in 14 GO terms (Table S4, Fig. 3C, Bonferroni p value of <0.05), and the translation process (Bonferroni p value = 9.54 × 10^−13^) is the most significant. The KEGG pathway enrichment analysis of ZFAS1-correlated PCGs showed that the pathway ribosome was significantly enriched (Bonferroni p value = 1.68 × 10^−16^). The functional analysis shows that ZFAS1 is implicated in ovarian cancer tumorigenesis via the positive regulation of protein-coding genes that affect translational and ribosome processes.

## Discussion

Conventionally, the study of gene regulation in biology has focused on protein-coding genes and miRNAs until the discovery of multiple functional regulatory lncRNAs. LncRNAs had increased disease- and tissue-specific expression levels than protein-coding genes, and their expression levels are more closely associated with its biological function^24^. Previous studies on tissue-specific lncRNAs in normal tissues and dysregulated lncRNA expression across various cancer types indicate that altered lncRNAs play critical roles in tumorigenesis^25^ via multiple cancer-related biological processes, such as apoptosis, cell cycle regulation, metastasis, and DNA damage response^26,27^. Furthermore, these dysregulated lncRNAs could mark the spectrum of tumor progression and have a great potential in the diagnosis and prognosis of cancer as novel independent molecular biomarkers^28,29^. Several dysregulated lncRNAs, such as HOTAIR and LSINCT5, are associated with ovarian cancer survival. However, to date, the expression profile-based prognostic lncRNA signatures for the prediction of chemotherapeutic sensitivity in ovarian cancer patients have not been developed.

In this study, a comprehensive analysis of lncRNA expression profiles in HGS-OvCa patients from TCGA was conducted. An eight-lncRNA predictive signature of chemotherapeutic sensitivity was identified via the logistic regression analysis. The increased expression levels of six lncRNAs were associated with the low probability of sensitivity, and three lncRNAs were correlated with the high probability of sensitivity. The eight-lncRNA signature is predictive of different molecular subtypes and better than the two published signatures. We also observed a close association between the eight-lncRNA signature and chemotherapeutic response within the TCGA dataset and four molecular subtypes. Furthermore, the eight-lncRNA signature is independent of other clinicopathological covariates, such as stage, grade, and molecular subtypes. To our knowledge, this study first showed the correlation of lncRNA expression profiles with chemotherapeutic sensitivity after platinum-based chemotherapy of HGS-OvCa.

To date, although an increased numbers of lncRNAs have been discovered and recorded in biological databases, such as GENCODE^30^, most of the lncRNAs were not functionally characterized. Only one of eight prognostic lncRNAs, namely, ZFAS1, has been reported as a prognostic biomarker and target of hepatocellular carcinoma^31^, colorectal cancer^32,33^, and gastric cancer^34^. According to the publication by Li *et al.*, ZFAS1 gene amplification is related with intrahepatic and extrahepatic metastasis and the poor prognosis of hepatocellular carcinoma, which functions as an oncogene by binding miR-150 and abolishing its tumor-suppressive roles^31^. ZFAS1 is significantly up-regulated in colorectal cancer tissues and may be an oncogene in colorectal cancer by the destabilization of p53 and interaction with CDK1/cyclin B1 complex, thus leading to cell cycle progression and apoptosis inhibition^32^. Furthermore, ZFAS1 expression is also overexpressed in gastric cancer, and its increased level is correlated with a shorter survival and poor prognosis and promotes the proliferation of gastric cancer cells by epigenetically repressing the KLF2 and NKD2 expression levels^34^. Our analysis identified the association of ZFAS1 with chemotherapy sensitivity in the training and validation datasets. The increased expression level of ZFAS1 was associated with the lower probability of sensitivity in patients with proliferative, mesenchymal, and differentiated subtypes. Further, based on *in vitro* experimental data, we concluded that the expression level of ZFAS1 could be regulated by cisplatin. Thus, ZFAS1 might play an important role in cisplatin resistance. Gene functional annotation revealed that ZFAS1 were likely involved in the translational process. To gain a deeper understanding of ZFAS1 roles and the effects of the other seven lncRNAs in response to chemotherapy in HGS-OvCa patients, the underlying regulatory mechanisms should be further explored.

Based on the molecular and genetic heterogeneity characteristics of ovarian cancer, we tested whether the prognostic value of the eight-lncRNA signature was independent of clinical characteristics. The multivariable logistic regression analysis revealed that the prognostic value of the eight-lncRNA signature was independent of stage, grade, and molecular subtypes. The eight-lncRNA signature might be used to update the current prognostic model and contribute to the strata of patients in future clinical trials.

The limitations of this study need to be presented. First, owing to the restricted availability of data, only a fraction of human lncRNAs (7740 out of 15000+) were included in our study. Second, although the biological functions of ZFAS1 have been inferred by gene functional annotation analysis, the mechanisms behind the predictive values of these eight lncRNAs in response to the chemotherapy of HGS-OvCas are still not clear, and their functional roles should be further explored in experimental studies. Finally, because other independent datasets are not available to validate our model, the significance and robustness of the eight-lncRNA signature for the prediction of chemotherapeutic sensitivity should be further investigated in clinical trials.

In summary, via probing and integrating available microarray expression data, our study presents a set of eight-lncRNA signature that is associated with chemotherapeutic sensitivity of HGS-OvCas. This signature might contribute to the identification of the low survival probability of patients who are likely to develop chemotherapy resistance. Gene functional annotation indicates that ZFAS1 might participate in the translational biological process. Our results confirmed that the identified signature lncRNAs might play potential roles in chemotherapeutic resistance mechanisms of HGS-OvCa tumors and are also considered as molecular diagnostic biomarkers and therapeutic targets in clinical practice.

## Materials and Methods

### Sources of data

Only HGS-OvCa specimens were used in the study that include the following datasets.

### Training dataset

The clinical information on HGS-OvCas (stages II, III, and IV and grades 2, 3, and 4) were obtained from Supplementary Table S1.2 (http://www.nature.com/nature/journal/v474/n7353/extref/nature10166-s2.zip) of TCGA’s publication^35^. Up to 258 of patients received at least six cycles of platinum treatment, and chemotherapeutic sensitivity information were used in this study. The clinical information of patients, including age, tumor stage and grade, chemosensitivity, chemoresponses, and molecular subtypes, are listed in Table 4 and Table S5.

**Table 4:** Patient characteristics of the training and validating datasets.

Characteristics	Training dataset	Validating dataset	P value^$^
Sample size	258	233	
Age, year mean (SD)	59.8 (11.2)	60.34 (9.9)	0.59
Histologic grade (%)			6.61 × 10^−12^
2	35 (13.6)	80 (34.3)	
3	223 (86.4)	138 (59.2)	
4	0	15 (6.4)	
Stage ^#^(%)			0.10
II	14 (5.4)	14 (6.0)	
III	206 (79.8)	199 (85.4)	
IV	38 (14.7)	20 (8.6)	
Platinum sensitivity (%)			0.02
Sensitive	190 (68.1)	185 (79.3)	
Response to therapy^&^			—
CR	194 (69.5)	0	
Non-CR	63 (22.6)	0	
Unknown	22 (7.9)	233 (100)	
Molecular subtypes			0.47
Proliferative	77 (29.8)	71 (30.5)	
Mesenchymal	51 (19.7)	39 (16.7)	
Immunoreactive	62 (24.0)	69 (29.6)	
Differentiated	68 (26.3)	54 (23.2)	

^#^Stage based on the International Federation of Gynecology & Obstetrics (FIGO).

^&^CR means the complete response, and Non-CR depicts a non-complete response, including partial response, progressive disease, and stable disease.

^$^p values for the difference between the derivation and validation cohorts were calculated using independent sample t-test (for age and height) and Chi square test (for histologic grade, stage, platinum sensitivity, response to therapy, and molecular subtypes).

LncRNA expression profiles by repurposing the probes from Affymetrix Human Exon 1.0 ST microarray of HGS-OvCa patients were downloaded from http://cistrome.org/lncRNA/lncRNA_data_repository.html ^36^. The probe sets that were not assigned for mRNAs but uniquely and perfectly mapped for noncoding RNA sequences that represent lncRNAs. The lncRNA expression levels were used as the background-corrected intensity of all probes mapped to this lncRNA. To reduce the heterogeneity of different batches and biological samples, the lncRNA expression value was standardized using the quantile-normalized method and Combat algorithm^37^. To reduce inaccurate annotations, the lncRNAs obtained from Du’s study and lncRNAs from the GENCODE project (http://www.gencodegenes.org/, release 25)^30^ were cross-referenced by Ensembl id and gene name. Finally, we obtained the expression profiles of 7739 lncRNAs. The lncRNA expression levels were modified with a mean of 0 and a standard deviation (SD) of 1.

### Validating datasets from GEO

Three datasets with the profiling data of gene expression obtained by using pretreatment biopsies in patients who received platinum-based chemotherapy and corresponding clinical data were downloaded from the GEO database (http://www.ncbi.nlm.nih.gov/geo/). All data were obtained with Affymetrix Human U133 Plus 2.0 arrays (Affymetrix). After the removal of the samples without progression-free survival information, a total of 233 advanced stage (stage > I) and high-grade (grade > 1) serous ovarian cancer patients were observed. A total of 141 patients from GSE9891 (24), 70 patients from GSE63885 (25), and 22 patients from GSE51373 (27) were included. The clinical information of the patients is listed in Table 4 and Supplementary Table S6.

The probe sets of Affymetrix Human U133 Plus 2.0 arrays that were not assigned for protein-coding transcripts and pseudogene transcripts but were uniquely and perfectly mapped for noncoding RNA sequences that were downloaded from http://cistrome.org/lncRNA/lncRNA_data_repository.html (file Array.probe.alignment/U133p2.lncRNA.uniq). Each lncRNA should include at least four probe mappings in the corresponding ncRNA entity. Up to 2654 probes corresponding to 2183 lncRNAs were left. The raw CEL files were downloaded from GEO, and all gene expression data were normalized with the MAS5 algorithm using the “simpleaffy” R Bioconductor package (http://www.bioconductor.org/packages/release/bioc/html/simpleaffy.html) with the mean expression focused at 600. The validating dataset was adjusted, which consists of three datasets for potential batch effects with the ComBat algorithm^37^. Furthermore, the probe-level expression profiles were converted into lncRNA-based expressions via probe merging with the collapse row function^38^. Finally, the lncRNA expression level of Affymetrix microarray datasets was scaled with a mean of 0 and an SD of 1.

### OVCA cultured cell lines

Forty-six ovarian cancer cell lines were subjected to treatment with cisplatin at the 50% growth inhibition concentration dosage. To explore transcriptomic responses to cisplatin, genome-wide expression changes were measured serially before and after cisplatin treatment. The gene expression was obtained using Human Gene ST 1.0 arrays (Affymetrix, Santa Clara, CA, USA), which was downloaded from the GEO with the accession number of GSE47856^23^. The cell lines with no less than three replicates (A2008, A2780, C13, CH1, DOV13, DOV13B, FU-OV-1, HeyA8, HeyC2, IGROV-1, OV90, OVCA420, OVCA429, OVCA433, OVCAR-8, PA-1, and TYK-nu) were tested in our study.

### Clinical outcomes

In the TCGA dataset, the platinum-free interval was the interval from the date of the last primary platinum chemotherapy to the date of recurrence, date of progression, or date of last follow-up if the patient is alive and did not experience recurrence. Platinum status was defined as resistant if the platinum-free interval was less than 6 months and was defined as sensitive if the platinum-free interval is 6 months or longer. However, no evidence on recurrence or progression existed, and the follow-up interval was at least 6 months from the date of the last primary platinum treatment. Patients who were monitored for less than 6 months from the date of the last primary platinum treatment and did not experience recurrence or progression were excluded from the analyses regardless of platinum status.

Chemotherapy response, the success of the primary therapy, was defined as the response to treatment determined after the primary surgery and subsequent adjuvant platinum chemotherapy. Following the primary therapy to determine the response, patients were evaluated with a combination of imaging (CT scan) and blood (CA125) tests. Patients with normalized CA125 and who did not show radiographic evidence of the disease were defined as complete responses^39^.

As for the dataset downloaded from GEO, platinum status was defined as resistant if the disease did not respond or progress during treatment or recur within 6 months of treatment^40^, and the status was defined as sensitive if the progression-free survival was 6 months or longer.

### Classification of HGS-OvCa subtypes

HGS-OvCas in the TCGA dataset were divided into proliferative, mesenchymal, immunoreactive, and differentiated subtypes according to the expression level of 100 genes by Verhaak *et al.* ^41^. Furthermore, the 100-gene set (Supplemental Table 7 from the publication by Verhaak *et al.*) was used to train support vector machines for the classification of samples in the validation datasets from GEO. The sample sizes for each subtype in the training and validating datasets are shown in Tables S5 and S6.

### Statistical analysis

To identify predictive lncRNAs, a univariate logistic regression analysis was performed to assess the relationship between the continuous expression level of each lncRNA and chemosensitivity. The lncRNAs with p values less than 0.003 were considered statistically significant and associated with chemosensitivity. Multivariate logistic regression was performed for the above-mentioned selected lncRNAs, and those lncRNAs with a p value of less than 0.01 were left for the predictive score calculation. The predictive score was computed to evaluate each patient’s probability of chemosensitivity according to the following formula:

1 $$predictive\,score(PS)=\sum _{i=1}^{n}(Ex{p}_{i}\,\ast \,Co{e}_{i}),$$

where n stands for the number of prognostic lncRNA genes in the model; Exp_i_ is the expression level of lncRNA_i_; Coe_i_ is the estimated regression coefficient of lncRNA_i_ in the multivariable logistic regression model. Patients who have higher predictive scores are expected to have a higher probability of response. Furthermore, the multivariate logistic regression analysis was conducted to test whether the predictive score was independent of clinical covariates.

Statistical computations were conducted using the R statistical software version 3.2.2^42^ with related packages or customized functions.

### Classifier performance evaluation

The area under the receiver operator characteristic curve (AUC) was used to evaluate the classification performance of the signatures according to their capability to distinguish between chemotherapeutic sensitivity and resistance. Moreover, AUC was calculated by R-package ROCR. The performance of our defined lncRNAs signature and two previously published signatures developed by Zhou *et al.* ^21^ and TCGA signature^35^ was compared.

### Coexpression and functional annotation

First, the expression profiles of 16936 PCGs in 258 HGS-OvCa patients were obtained from Du’s study^36^. The biological functions of lncRNAs are associated with the coexpressed PCGs^43^. Thus, the expression correlation between lncRNAs and PCGs with the expression profiles of paired lncRNA and PCG was tested. The PCGs were lncRNA correlated if their correlation coefficients with this lncRNA were not less than 0.4.

The GO biological process (GOTERM-BP-ALL) and Kyoto encyclopedia of genes and genomes (KEGG) pathway enrichment analyses of the PCGs coexpressed with prognostic lncRNAs were performed to predict the function of prognostic lncRNAs via the DAVID annotation tool (http://david.abcc.ncifcrf.gov/) with the functional annotation clustering option^44^. The enriched GO terms and KEGG pathway with a Bonferroni p value of <0.05 were considered as a potential function of prognostic lncRNAs. The significantly enriched GO terms with a similar function were visualized using the Enrichment Map Plugin in Cytoscape^45^.

## Electronic supplementary material

Supplementary Table S2

Supplementary Tables
