# Supplementary material for: Long noncoding RNA expression signature to predict platinum-based chemotherapeutic sensitivity of ovarian cancer patients
Source: Sci Rep. 2017 Feb 2;7:18. doi: 10.1038/s41598-017-00050-w (PMC5428368; doi:10.1038/s41598-017-00050-w)
Supplement: Supplementary file 1 — Supplementary Table S2 [file 41598_2017_50_MOESM1_ESM.doc]

**Long noncoding RNA expression signature to predict platinum-based chemotherapeutic sensitivity of ovarian cancer patients**

Rong Liu^1,2^, Ying Zeng^1,2^, Cheng-Fang Zhou^1,2^, Ying Wang^3^, Xi Li^1,2^, Zhao-Qian Liu^1,2^, Xiao-Ping Chen^1,2^, Hong-Hao Zhou^1,2^*, Wei Zhang^1,2^*

1. Department of Clinical Pharmacology, Xiangya Hospital, Central South University, Changsha 410008; P. R. China.
2. Institute of Clinical Pharmacology, Central South University; Hunan Key Laboratory of Pharmacogenetics, Changsha 410078; P. R. China.
3. The Affiliated Cancer Hospital of XiangYa School of Medicine, Central South

University, Changsha, Hunan 410014; P. R. China.

Table S2. Expression values of ZNFS1 in seventeen cell lines treated with either cisplatin or not.

| **Cell line** | **Sample size (test:control)** | **P value *** | **Direction^#^** |
| --- | --- | --- | --- |
| A2008 | 3:3 | 1.39×10^-2^ | Up |
| HeyA8 | 3:3 | 9.57×10^-3^ | Up |
| HeyC2 | 3:3 | 1.75×10^-2^ | Up |
| OVCA429 | 3:3 | 2.49×10^-2^ | Down |
| C13 | 3:3 | 5.22×10^-2^ | _ |
| A2780 | 3:3 | 2.01×10^-1^ | _ |
| CH1 | 3:3 | 1.00×10^0^ | _ |
| DOV13 | 3:3 | 1.13×10^-1^ | _ |
| DOV13B | 3: 3 | 9.30×10^-2^ | _ |
| FU-OV-1 | 3:3 | 8.09×10^-2^ | _ |
| IGROV-1 | 3:3 | 2.03×10^-1^ | _ |
| OV90 | 3:3 | 2.28×10^-2^ | _ |
| OVCA420 | 3:3 | 8.88×10^-2^ | _ |
| OVCA433 | 4:4 | 6.48×10^-2^ | _ |
| OVCAR-8 | 3:3 | 3.91×10^-1^ | _ |
| PA-1 | 4:3 | 6.59×10^-1^ | _ |
| TYK-nu | 3:3 | 4.58×10^-1^ | _ |

*P values were calculated by independent two-tailed t test.

# Up stands for up regulated in the group treated with cisplatin, down stands for down regulated in the group treated with cisplatin, “-“stands for no significant difference.
